# Caesarean delivery of first prediagnosed COVID-19 pregnancy in Nigeria

**DOI:** 10.11604/pamj.2020.36.100.23892

**Published:** 2020-06-16

**Authors:** Christian Chigozie Makwe, Kehinde Sharafadeen Okunade, Muyiwa Kayode Rotimi, Oluwayemisi Esther Ekor, Olalekan Gabriel Oyeleke, Qazeem Oladele Bello, Ayodeji Ayotunde Oluwole, Iorhen Ephraim Akase, Beatrice Nkoli Ezenwa, Iretiola Bamikeolu Fajolu, Rotimi Williams Dada, Yewande Oshodi, John Olutola Olatosi, Olabisi Oluranti Opanuga, Sunday Omilabu, Veronica Chinyere Ezeaka, Bosede Bukola Afolabi

**Affiliations:** 1 Department of Obstetrics and Gynaecology, College of Medicine, University of Lagos, Lagos, Nigeria,; 2 Department of Anaesthesia, College of Medicine, University of Lagos, Lagos, Nigeria,; 3 Department of Anaesthesia, Lagos University Teaching Hospital, Lagos, Nigeria,; 4 Department of Paediatrics, Lagos University Teaching Hospital, Lagos, Nigeria,; 5 Department of Nursing Services, Lagos University Teaching Hospital, Lagos, Nigeria,; 6 Infectious Diseases Unit, Department of Medicine, Lagos University Teaching Hospital, Lagos, Nigeria,; 7 Neonatology Unit, Department of Paediatrics, College of Medicine, University of Lagos, Lagos, Nigeria,; 8 Department of Psychiatry, College of Medicine, University of Lagos, Lagos, Nigeria,; 9 Pharmacy Department, Lagos University Teaching Hospital, Lagos, Nigeria,; 10 Department of Medical Microbiology and Parasitology, College of Medicine, University of Lagos, Lagos, Nigeria

**Keywords:** COVID-19, pregnancy, Nigeria, caesarean section, SARS-CoV-2, delivery

## Abstract

The COVID-19 pandemic is currently causing widespread infection and deaths around the world. Since the identification of the first case in Nigeria in February 2020, the number of confirmed cases has risen to over 9,800. Although pregnant women are not necessarily more susceptible to infection by the virus, changes to their immune system in pregnancy may be associated with more severe symptoms. Adverse maternal and perinatal outcomes have been reported among pregnant women with COVID-19 infection. However, literature is scarce on the peripartum management and pregnancy outcome of a pregnant woman with COVID-19 in sub-Saharan Africa. We report the first successful and uncomplicated caesarean delivery of a pregnant woman with COVID-19 infection in Nigeria.

## Introduction

The novel coronavirus (COVID-19) was first identified in Wuhan City, Hubei province of China, towards the end of 2019 [1]. The COVID-19 pandemic is currently causing widespread infection and deaths around the world; with growing concern in the medical community [2]. Globally, the number of confirmed cases has exceeded 5 million with hundreds of thousands of death [2]. Since the identification of the first case in Nigeria in February 2020, the number of confirmed cases has risen to over 9,855 as of May 30, 2020 [3]. The confirmatory test for COVID-19 infection is real-time reverse-transcription polymerase chain reaction (RT-PCR) of nasopharyngeal and/or oropharyngeal swabs [4]. The virus, also known as SARS-CoV-2, is transmitted via droplet inhalation to close contacts of affected individuals and through fomites [5]. The risk of infection transmission increases with the duration of close contact with a COVID-19 infected individual; especially a person who has symptoms. Although the possibility of vertical transmission exists [6, 7], this has not been fully established [8,9]. COVID-19 infection causes respiratory tract illness ranging in severity from the mild common cold to severe and fatal illness.

The disease seems to be more severe in older people, the immunosuppressed and those with long-term conditions such as diabetes, cancer and chronic lung disease [9]. Although pregnant women are not necessarily more susceptible to viral illness, changes to the immune system in pregnancy can be associated with more severe symptoms such as pneumonia [6]. This is particularly true towards the end of pregnancy and during childbirth. However, available literature suggests that the clinical manifestation of COVID-19 in pregnant women is similar to non-pregnant sufferers [6, 8]. Though a novel virus, scientific publications have reported possible detrimental effects of COVID-19 infection during pregnancy and childbirth such as maternal pneumonia and maternal death, intrauterine fetal death/stillbirth, preterm birth, neonatal pneumonia/sepsis, disseminated intravascular coagulopathy and neonatal death [6-9]. Literature is scarce on peripartum management of a pregnant woman with COVID-19 and her exposed newborn in sub-Saharan Africa. We report the first uncomplicated caesarean delivery in a pregnant woman with COVID-19 infection in Nigeria.

## Patient and observation

A 37-year-old woman, gravida 5 para 2, at 38 weeks´ gestation was referred to Lagos University Teaching Hospital (LUTH) for caesarean delivery. Prior to her referral, she had been scheduled for an elective caesarean section at 38 weeks´ gestation due to two previous caesarean sections and a previous abdominal myomectomy. Her antenatal care was essentially uneventful and ultrasonography confirmed a singleton pregnancy. She had nasopharyngeal and oropharyngeal swabs collected for RT-PCR test, after close contact with a confirmed case of COVID-19. She had no symptoms associated with COVID-19 infection and no history of recent travel. However, she complained of abdominal pain and intermittent uterine contractions. She was commenced on oral nifedipine for tocolysis. Her laboratory test confirmed COVID-19 infection after 48 hours and she was referred to LUTH immediately. Although LUTH has assigned wards for the management of patients with COVID-19, there were no dedicated spaces for infected pregnant women and their neonates. However, there was a dedicated operating theatre with a safe corridor to one of the COVID-19 isolation wards. The woman was admitted in a private room at the isolation ward prior to the caesarean section.

A multidisciplinary team of obstetricians, anaesthetists, neonatologists, nurses, psychiatrists and infectious disease experts were assembled and simulation sessions were undertaken based on the established infection prevention and control (IPC) guidelines. All staff caring for the woman wore appropriate personal protective equipment (PPE). Her laboratory results (Table 1) were unremarkable except for lymphopenia (a common laboratory finding in patients with COVID-19) [6, 9] and hypokalaemia. The correction of hypokalaemia was commenced with parenteral potassium infusion. She was appropriately counselled and reassured to calm her perioperative anxiety. A remote consent was taken via video call, after sharing appropriate information material via WhatsApp messaging. On admission day 2, she was transported to the operating theatre while observing all the necessary IPC measures. After signing a written informed consent, she had a lower segment caesarean section and bilateral tubal ligation performed under spinal anaesthesia. She was delivered of a full-term male baby weighing 2600g with Apgar score 9 and 10 in 1 and 5 minutes, respectively. Intraoperative oxygen saturation was maintained at a minimum of 98%, intraoperative infusion volume was 1000mL and estimated blood loss was 600mL.

**Table 1:** clinical laboratory result

Admission		Day 1	Day 4
Variables	Reference range	Value	Value
Sodium (mmol/L)	135 - 145	133	148‡
Potassium (mmol/L)	3.5 - 5.1	2.7†	2.89†
Chloride (mmol/L)	98 - 110	103	112‡
Bicarbonate (mmol/L)	22 - 30	19†	15†
Creatinine (µmol/L)	39 - 91	46	54
Urea (mmol/L)	2.5 - 6.4	2.5	3.6
Anion gap (mmol/L)	3 - 15	11	21
Haemoglobin (g/dl)	11.0 - 16.0	12.3	11.5
Haematocrit (%)	33 - 48	36.2	34.2
Platelets (x 10^9^/L)	150 - 450	163	163
White cell count (x 10^9^/L)	4.0 - 11.0	4.63	13.84‡
Neutrophils (x 10^9^/L)	2.0 - 7.5	3.29	12.25‡
Lymphocytes (x 10^9^/L)	1.0 - 4.0	0.78†	1.08
Monocytes (x 10^9^/L)	0.0 - 1.0	0.52	0.48
Eosinophils (x 10^9^/L)	0.0 - 0.4	0.01	0.01
Basophils (x 10^9^/L)	0.0 - 0.1	0.03	0.02

† The value in patient below normal range

‡ The value in patient below normal range

She had prophylactic intravenous tranexamic acid 1g and carbetocin 100μg. The mother and baby were nursed in the recovery room of the operating theatre and the immediate postnatal period was uneventful. The newborn was commenced on breast milk substitute until the mother was able to breastfeed the baby on demand. On day 3 after delivery, the mother and her newborn were transferred back to the isolation ward. The nasopharyngeal swabs collected from the neonate (at birth and 48 hours after birth) were all negative for COVID-19 infection. Both mother and baby remained asymptomatic during the postpartum period. On day 15 after delivery, the mother met the criteria for discharge because she had remained asymptomatic for more than 14 days after her initial positive result [4]. After appropriate counselling, the mother and her baby were discharged home and scheduled for a follow-up visit in 2 weeks. Throughout the period of care, contact between staff and patient was minimised and she wore a surgical mask all the time. Telephone conversations were frequently used for communication between the woman and the multi-disciplinary team members (obstetrician, anaesthetist, neonatologist and psychiatrist). Fourteen days after the delivery, all her caregivers remained well.

## Discussion

This case describes an uncomplicated caesarean section in a woman with COVID-19 infection in a low resource setting. Her pregnancy was considered high-risk due to the three previous uterine surgeries and the potential risk of uterine rupture. Despite the hypokalaemia, the multidisciplinary team decided to proceed with the caesarean delivery on the second day of admission because of the uterine contractions and risk of uterine rupture. She had a successful and uncomplicated caesarean section. A successful pregnancy outcome in the management of a woman with COVID-19 infection requires a multidisciplinary team approach and facility preparedness [10]. There is, therefore, a need to allocate adequate resources for the management of pregnant women with COVID-19 and their exposed neonates. The index case captures the apprehension, challenges and fear faced by the COVID-19 positive pregnant woman, her newborn and the healthcare providers. There is a need for proper planning, coordination and communication among the healthcare staff. During this COVID-19 pandemic, the role of telecommunication in medicine is essential and health institutions need to build the necessary infrastructure and capacity for telemedicine in this COVID era, particularly in low- and middle-income countries. Laboratory testing capacity and the turn-around time for RT-PCR testing also need to be improved, particularly in the management of obstetric emergencies, where a 48-hour turn-around time might be detrimental for the mother and her baby. The possible role of COVID-19 rapid diagnostic tests need further evaluation and validation in the case management of pregnant women with suspected COVID-19 infection; especially in the absence of RT-PCR test capabilities.

## Conclusion

Although the setting was less than ideal, we ensured the provision of safe and effective care for the woman and her newborn, including her mental health and emotional wellbeing. The necessary precautions were taken to prevent dissemination of infection among patients and healthcare workers. After the caesarean delivery, there was no evidence of infection transmission to caregivers.
